# Look-Locker T1 relaxometry and high-resolution T2 in the evaluation of lung lesions: a single-center prospective study

**DOI:** 10.1590/0100-3984.2024.0033

**Published:** 2024-09-30

**Authors:** Danilo Tadao Wada, Li Siyuan Wada, Camila Vilas Boas Machado, Mateus Repolês Lourenço, Tales Rubens de Nadai, Federico Enrique Garcia Cipriano, Alexandre Todorovic Fabro, Marcel Koenigkam-Santos

**Affiliations:** 1 Faculdade de Medicina de Ribeirão Preto da Universidade de São Paulo (FMRP-USP), Ribeirão Preto, SP, Brazil.; 2 Faculdade de Medicina de Bauru da Universidade de São Paulo (FMBRU-USP), Bauru, SP, Brazil.

**Keywords:** Solitary pulmonary nodule, Magnetic resonance imaging, Lung neoplasms, Lung, Nódulo pulmonar solitário, Ressonância magnética, Neoplasias pulmonares, Pulmão

## Abstract

**Objective:**

To explore the feasibility of two magnetic resonance imaging (MRI) sequences—high-resolution T2-weighted (HR T2) and Look-Locker T1 (LL T1) relaxometry—for the investigation focal lung lesions (FLLs). As a secondary objective, we analyzed the diagnostic accuracy of these sequences.

**Materials and Methods:**

This was a prospective observational study involving 39 subjects with FLLs scanned in a 1.5-T MRI system with LL T1 relaxometry and HR T2 sequences focused on the FLL region, in addition to a conventional protocol. All images were evaluated by two radiologists, working independently, who were blinded to other findings.

**Results:**

Most of the examinations (31 of the LL T1 relaxometry sequences and 36 of the HR T2 sequences) were of adequate diagnostic quality. Nondiagnostic examinations were considered so mainly because of limited coverage of the sequences. Of the FLLs studied, 19 were malignant, 17 were benign, and three were excluded from the accuracy analysis because there was no definitive diagnosis. Although LL T1 relaxometry could not distinguish between benign and malignant lesions, the signal intensity at its first inversion time (160 ms) differed between the two groups. The HR T2 sequence was considered the best sequence for assessing specific morphological characteristics, especially pseudocavities and pleural tags. We found that MRI showed better accuracy than did computed tomography (86% vs. 74%).

**Conclusion:**

Both MRI sequences are feasible for the evaluation of FLLs. Images at 160 ms of the LL T1 relaxometry sequence helped distinguish between benign and malignant lesions, and the HR T2 sequence was considered the best sequence for evaluating specific morphological characteristics.

## Abstract (pt)

**Objetivo:**

Explorar a viabilidade de imagens de alta resolução T2 (T2 AR) e relaxometria T1 Look-Locker (T1 LL) para lesões pulmonares focais (LPFs). Como objetivo secundário, analisamos a precisão diagnóstica dessas sequências.

**Materiais e Métodos:**

Este é um estudo observacional prospectivo com 39 sujeitos com LPFs examinados em um sistema de ressonância magnética 1.5T com imagens T1 LL e T2 AR focadas na região das LPFs, além de um protocolo convencional. As imagens foram avaliadas por dois radiologistas independentes e cegos para o estudo. Imagens de tomografia computadorizada estavam disponíveis, mas foram avaliadas sem conhecimento dos outros resultados.

**Resultados:**

A maioria dos exames apresentou qualidade diagnóstica adequada em ambas as sequências (T1 LL em 31 exames e T2 AR em 36). Exames considerados não diagnósticos estavam principalmente relacionados à cobertura limitada das sequências. Das LPFs estudadas, 19 eram malignas, 17 eram benignas e três casos foram excluídos da análise de precisão de malignidade por falta de um diagnóstico definitivo. A relaxometria T1 LL não conseguiu distinguir entre lesões benignas e malignas, mas a análise da intensidade do sinal do primeiro tempo de inversão (160 ms) diferiu entre os grupos. A T2 AR foi considerada a melhor sequência para avaliar características morfológicas específicas, especialmente pseudocavidades e apêndices pleurais. A ressonância magnética teve melhor precisão em comparação com a tomografia computadorizada (86% e 74%, respectivamente).

**Conclusão:**

Ambas as sequências são viáveis na avaliação de LPFs. Imagens a 160 ms da sequência T1 LL ajudaram a distinguir lesões benignas de malignas, e a T2 AR foi considerada a melhor sequência na avaliação de algumas características morfológicas específicas.

## INTRODUCTION

Imaging examinations play a fundamental role in all stages of evaluation of lung nodules, masses, and cancer, including screening, characterization, staging, treatment response assessment, and follow-up. Computed tomography (CT) is the imaging examination of choice in clinical practice, being the most sensitive for detecting focal lung lesions (FLLs). However, CT still does not have the desired specificity to avoid the follow-up or even biopsy and resection of benign lesions, and the false-positive rate is a matter of concern, even in lung cancer screening programs. Lung nodules and masses are highly prevalent findings on chest imaging^(1,2,3,4)^. As previously demonstrated in the National Lung Cancer Screening Trial^(5,6)^, as well as in the Nederlands-Leuvens Longkanker Screenings Onderzoek (Dutch-Belgian Lung Cancer Screening Trial) and other studies^(7,8,9)^, they are also the most common representation of malignant neoplasms in the lungs, whether primary or metastatic. Therefore, the differentiation between pulmonary lesions that represent benign changes and those that are malignant is of great importance in clinical practice.

Magnetic resonance imaging (MRI) is a method with higher contrast resolution and multiparametric capability. Therefore, it can add important information not only in the morphological assessment but also in the functional, physiological, pathophysiological, and molecular characterization, through the use of specific sequences. There have always been significant limitations for its use in lung cancer screening, including the low proton density in lung structures, magnetic susceptibility artifacts resulting from differences between tissues and air, and physiological movement artifacts (resulting from breathing and heartbeats). However, the development of new image acquisition techniques and equipment has increased its potential for use in different scenarios^(10,11,12)^, including as a complement to CT in the characterization of FLLs, as recommended by the Fleischner Society^(13)^.

The MRI evaluation of any given organ requires specific sequences optimized to provide the greatest diagnostic power. In addition, specific protocols are employed to assess each specific situation, leading to a more accurate diagnosis. There are numerous existing sequences and many modifiable variables in each one, allowing many combinations. Therefore, there are many sequences used specifically in the evaluation of certain organs. Some of those have not been tested in the evaluation of FLLs.

In cardiac examinations, T1-weighted sequences with inversion recovery have routinely been used as scout images to determine the correct nulling time of the myocardial signal in sequences that assess fibrosis and inflammation in the heart^(14,15,16,17)^. The most widely available of such sequences is called T1 Look-Locker (LL T1) relaxometry. Given its multiple inversion time characteristic, it has a quantitative nature that allows the calculation of T1 re-laxometry of tissues.

When MRI is used in colorectal cancer staging and in the screening of focal prostatic lesions, T2-weighted sequences are considered the most relevant^(18,19,20,21)^, and it is recommended that they be performed with high-resolution techniques to obtain information such as transmural extension of colorectal cancer and the identification of focal lesions in the prostate, given that it is such a small organ. Therefore, it is seen as a sequence of great power for morphological characterization.

The present study aimed to explore the feasibility of two new MRI sequences for the evaluation of FLLs: an LL T1 relaxometry sequence for native T1 relaxometry based on inversion recovery; and a high-resolution T2-weighted (HR T2) morphological sequence. We also evaluated the capability of these sequences to discriminate between benign and malignant FLLs.

## MATERIALS AND METHODS

### Study population

This was a prospective observational study. We recruited consecutive patients who underwent MRI between October 2017 and December 2019. All participants or their legal guardians gave written informed consent, and the study was approved by the local research ethics committee. Patients were recruited if they had been referred for a CT-guided lung biopsy and had undergone an MRI examination before (on the day of) the biopsy; if they had been assessed in a multidisciplinary discussion involving oncology, thoracic surgery, radiology, and pathology teams, with an MRI examination indicated as part of the clinical routine; or if they had been scheduled to undergo chest CT with a Swensen protocol for lung nodules, with MRI examinations being scheduled on the basis of scanner availability. All patients underwent the MRI examinations voluntarily, at no cost to them and without interference in the management of their cases. The examinations were scheduled during the investigation of the FLLs only if they would not delay the diagnosis.

The following inclusion criteria were applied: having a lung nodule or mass; being under investigation, follow-up, or treatment at our institution; being between 18 years and 80 years of age; and having undergone multidetector CT with thin volumetric slices (< 2 mm) for morphological correlation. Patients who were unable to complete the chest MRI were excluded, as were those in whom the intravenous injection of macrocyclic gadolinium-based contrast media was contraindicated, those with lesions larger than 5 cm, and those with lesions that had been treated previously (with systemic therapy or radiotherapy). After applying the inclusion and exclusion criteria, we numbered the remaining cases successively, from first to last, based on the MRI examination date.

### MRI protocol

All participants underwent chest MRI in a 1.5-T scanner (Achieva; Philips Medical Systems, Best, The Netherlands) with a phased-array surface coil, in the supine position. In addition to the routine chest MRI protocol of our institution, LL T1 relaxometry and HR T2 sequences were acquired.

For the LL T1 relaxometry, a peripheral pulse unit (PPU) was used as a heartbeat triggering mechanism. Technically, the acquisition requires synchronization with heartbeats, which can be done by gating the examination with an electrocardiogram or a PPU. Although simple, the synchronization is more complex when achieved by electrocardiogram than when achieved by PPU, the former requiring disposable electrodes and more time for patient preparation (e.g., the patient may need to be shaved), as well as having limitations related to low voltage in patients with lung problems (e.g., hyperinflation by lung emphysema), which can impede the detection of the cardiac rhythm. In contrast, the use of a PPU is more straightforward and is therefore routinely employed to perform magnetic resonance angiography and cerebrospinal fluid flow sequences.

The HR T2 sequence was acquired during free breathing with five signal averages and respiratory gating through an external trigger. The LL T1 relaxometry and HR T2 sequences were both acquired before intravenous contrast media injection. Specific sequence parameters and details are shown in Table 1.

**Table 1:** Summary of the parameters of the study sequences.

Sequence	Sequence parameters	Plane, slice position, and coverage	Breath hold	Approximate scan time (mm:ss)
IR-based LL T1 relaxometry	TE: 2.9 ms; TR: 8 ms; FA: 12º; trigger times: 160, 184, 208, 232, 256, 281, 305, 329, 353, 377 , 401, 42 5, 45 0, 474, 49 8, 522, 546, 570, 595, 619, 643, 667, and 691 ms; NSA: 1; peripheral pulse gated	Axial; slices: 1, at the FLL level; FOV: 350 mm; slice thickness: 10 mm; acquisition matrix: 144 × 141; reconstruction matrix: 256 × 256	Breath hold at full inspiration; cardiac triggered through a PPU	00:16
Turbo spin-echo HR T2	TE: 90 ms; TR: 1600 ms; FA: 90º; NSA: 5	Axial; slices: 15, centered at the FLL level; FOV: 400 mm; slice thickness: 3.5 mm; inter-slice gap: 0.3 mm; acquisition matrix: 584 × 289; reconstruction matrix: 640 × 640	Free breathing	05:30

TE, echo time; TR, repetition time; FA, flip angle; NSA, number of signal averages; FOV, field-of-view.

### Image evaluation

The images were transferred to an image storage and distribution server and analyzed on a dedicated workstation running the MacOs operating system, version 10.13.6 (Apple, Cupertino, CA, USA) with the free medical image viewer Horos, version 3.3.6. Horos is open-source software, distributed free of charge under a Lesser General Public License via the website Horosproject.org (Nimble Co LLC, dba Pureview, Annapolis, MD, USA).

The CT and MRI examinations were evaluated by two experienced thoracic radiologists with 5 and 15 years of experience, respectively, working independently. The more experienced of the two was certified by the European Society of Thoracic Imaging and European Society of Radiology. The evaluators were blinded to the other imaging findings, histopathological results, and final diagnosis.

The feasibility of each sequence was analyzed on a per-examination basis. The images deemed to be of poor image quality (e.g., degenerated by artifacts that precluded the proper evaluation of the FLL) were excluded from the evaluation.

The segment in which each lesion was located was noted (according to Boyden’s modified lung segmentation), as were the morphological characteristics (size, nature, type, and contours) and presence of additional findings (pseudocavity, air bronchogram, or pleural tag). Other parameters outside the main scope of the study (type of enhancement, signal intensity on T1- and T2-weighted images, signal on a diffusion-weighted imaging sequence, apparent diffusion coefficient, and contrast perfusion pattern) were also evaluated. Morphologically, the lesions were classified by using the descriptors proposed by the Fleischner Society^(22,23)^: for nature—solid, nonsolid (ground-glass only), or partially solid (when there was a solid lesion associated with a ground-glass component); for type—nodule, mass, consolidation, or other; and for contours—regular, lobulated, spiculated, or irregular.

In the qualitative analysis of the morphological characteristics, the sequence that best demonstrated the contours, as well as the presence of ground-glass components, pseudocavities, air bronchograms, and pleural tags, was chosen by consensus among the radiologists.

For the quantitative parameters, the lesion size was considered the largest linearly measurable value in the axial plane, without including areas of contour irregularity or spikes, as suggested in the Fleischner Society guideline for nodule measurement^(24)^. An elliptical region of interest was drawn, encompassing at least half of the area of the lesion in the slice, after which the mean signal intensity of the pixels was noted. The values and standard deviations (SDs) of signal intensity measured in the air were also recorded in order to evaluate the quality of the sequences on the basis of the signal-to-noise ratios^(25)^.

For each lesion, the native T1 time was calculated by fitting the measured mean signal intensity of each region of interest in each inversion time. When using inversion recovery techniques to calculate native T1, images are acquired repeatedly after an initial inversion pulse, allowing the plotting of a recovery curve^(26)^. Each of the acquired images has a well-defined inversion time. One of the positive characteristics of the sequence is the generation of multiple images, allowing more precise fitting for the calculation of T1. However, the sequence also has disadvantages. The radiofrequency pulse employed to acquire the data also affects the T1 recovery curve, generating an apparent Tl (T1*), which is not the same as T1 without the disturbance for inversion recovery. The T1 relaxation time can be estimated using approximations such as *T1* = (*B/A* – 1)T1*, where B and A are constants. Another detail is that if there is no complete relaxation between the different acquisition cycles of the sequence, artifacts measuring the T1 time related to heart rate appear. In this situation, measurements will be imprecise in tissues with longer T1 times, becoming even more imprecise as heart rates get higher unless the variation is compensated for at the time of fitting.

After the images in all sequences had been evaluated, the lesions were classified, according to the degree of suspicion for neoplastic etiology, as probably benign or not typically benign/indeterminate. In the assessment of CT images, the location and morphological characteristics (size, density, morphology, contours, and type of enhancement) of the lesion, as well as the presence of additional findings (pseudocavities, air bronchograms, or pleural tags) were also noted.

### Malignancy assessment

To assess the malignancy characterization capability of each sequence studied, we separated the patients into two groups: those ultimately diagnosed with a benign lesion (benign group); and those ultimately diagnosed with a malignant lesion (malignant group). The final diagnosis was based on the pathology examination or on the multidisci-plinary consensus documented in the electronic medical record. Lesions that met stability criteria in imaging examinations for a period of at least 24 months were considered benign, whereas those with highly suspicious imaging and clinical data consistent with cancer (such as the progression of suspicious lesions to secondary involvement, with a concomitant increase in tumor serum markers) were considered malignant. For this accuracy analysis, cases in which there was no definitive diagnosis were excluded.

### Statistical analysis

Descriptive statistics and graphical data were analyzed with GraphPad Prism, version 7.0.5 (GraphPad Software, San Diego, CA, USA). All data were organized and analyzed on a personal computer, using Microsoft Excel for Microsoft Office 365. The distribution of each data parameter was tested for normality by using the Kolmogorov-Smirnov test. To compare normal distribution parameters between two independent groups, we used the unpaired t-test. For data without a normal distribution, we used the nonparametric Mann-Whitney U test. Categorical variables were analyzed with the chi-square test. Correlations were tested with Spearman’s correlation test. We considered a significance level of 95%, with values of *p* < 0.05 being considered statistically significant.

## RESULTS

### Study population

Forty-five subjects were recruited and started an MRI examination. Two subjects failed to complete the examination, and one had an incomplete examination due to contraindication for the use of contrast media (acute renal failure). Three other patients met the exclusion criteria: one because of age (83 years), one because of lesion size (63 mm), and one because the indication for the examination was a pseu-dotumor (focal herniation of abdominal fat through a diaphragmatic hernia, mimicking a pulmonary mass).

**Figure 1:**
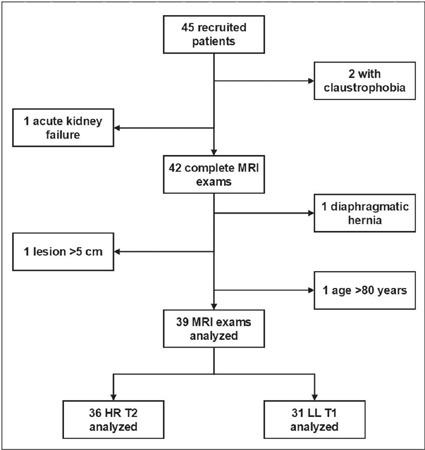
Flow chart of the patient selection process.

Of the initial 45 subjects, 27 had examinations ordered directly by an attending clinician, 16 were recruited on the day they appeared for a CT-guided percutaneous biopsy, and two were approached during a CT examination with the Swensen protocol. The final sample comprised 39 MRI examinations submitted to technical evaluation of the sequences (Figure 1). The clinical and epidemiological characteristics of the 39 study subjects are shown in Table 2. All of the examinations evaluated were performed without complications, and no adverse reactions related to intravenous contrast medium injections were observed.

**Table 2:** Demographic and clinical characteristics of the patients, by group.

Characteristic	Total (N = 39)	Malignant FLL (n = 19)	Benign FLL (n = 17)	P*
Age (years), mean ± SD	61.7 ± 13	64.6 ± 9.6	58.1 ± 15.7	0.310
Male, n (%)	22 (56.4)	12 (63.2)	8 (47.0)	0.526
Known malignancy, n (%)	18 (46.2)	10 (52.6)	6 (35.3)	0.478
Smoker, n (%)	26 (66.7)	14 (73.7)	9 (52.9)	0.344
Smoking history (pack-years), mean ± SD	58.4 ± 62.6	74.0 ± 75.8	35.9 ± 24.2	0.300

*Malignant vs. benign.

### LL T1 relaxometry and HR T2 sequence viability

In two of HR T2 sequences, the FLLs were not covered (Figure 1) in the acquisition programmed by the MRI technician, because of the limited number of slices focused on the lesion. In another one of the examinations, performed at the beginning of the protocol adjustment period, the HR T2 sequence was accidentally performed after intravenous injection of the contrast medium and was therefore also excluded from the analysis. Technical errors occurred in examination numbers 1, 19, and 27. Therefore, it was possible to assess lesions in the HR T2 sequence in 36 (92.3%) of the 39 examinations performed. In the HR T2 sequences, the mean signal-to-noise ratio was 11.6, compared with 883.4 for the conventional T2-weighted sequence, showing that the noise level was higher in the HR T2 sequence.

The acquisition of LL T1 relaxometry sequences presented more technical difficulty in its implementation than did that of the HR T2 sequences. Six patients were excluded from the analysis because of technical errors or artifacts that prevented the assessment of the lesions: in one case, the sequence was not acquired; in two cases, the lesions were not covered at the programmed acquisition, probably because of an error in the programming of the acquisition field or because there were different levels of inspiration during breath-hold maneuvers; in one case, the LL T1 relaxometry sequence was acquired without the use of a PPU, making it impossible to synchronize with the heartbeat; in another case, the breath-hold was not satisfactory, causing the nodule not to be seen in the last images of the acquisition; and in the last of those six cases, there were aliasing artifacts in the image, folding over the nodule. The mean size of the lesions in which errors occurred was 15 mm, in line with the mean of the sizes of all lesions (18 mm). In chronological order, the examinations in which the assessment of the LL T1 relaxometry sequence was excluded from the analysis were numbers 1, 3, 13, 17, 28, and 34, showing a uniform distribution throughout the data collection period. In total, we were able to analyze the LL T1 relaxometry in 33 (79.5%) of the 39 acquisitions. In two of those 33 cases, because the heart rates were higher, it was necessary to use a smaller number of inversion times, a known situation in which the accuracy of the measurement in tissues with longer T1 times is compromised.

**Figure 2:**
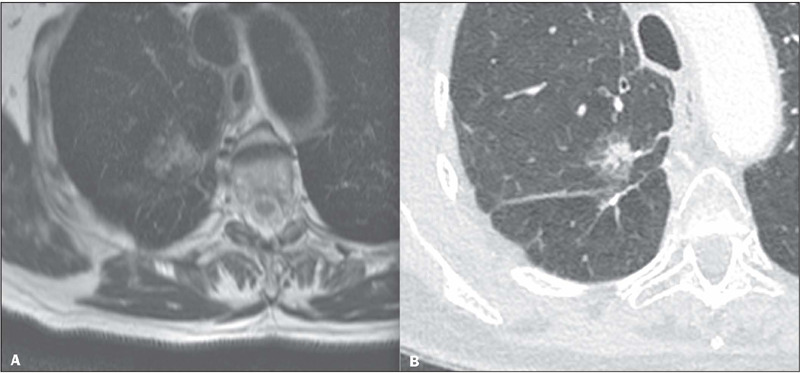
Partially solid nodule on an HR T2 sequence **(A)** and on CT **(B).**

As evaluated by both readers, the contours of FLLs were best depicted in HR T2 sequences in 32 (82.0%) of the 39 cases, followed by contrast-enhanced T1-weighted sequences in seven (17.9%). Regarding the detection of specific characteristics of the lesions, the imaging showed a ground-glass component in one case (Figure 2), a pseudocavity in one, air bronchograms in three, and pleural tags in seven. Those characteristics were best evaluated in the HR T2 sequences in one, one, zero, and six cases, respectively, the remainder having been considered to be better characterized in the contrast-enhanced T1-weighted sequences, as shown in Table 3.

**Table 3:** Prevalence of specific morphological features in FLLs, with both imaging modalities, and identification of the MRI sequence that provided the optimal visualization.

Feature	CT/MRI (n/n)	HR T2 (n)	Volumetric CE T1 (n)
Pseudocavity	1/1	1	0
Air bronchogram	3/3	0	3
Pleural tag	13/7	6	1

CE T1, contrast-enhanced T1-weighted (sequence).

### Malignancy characterization

Three patients (cases 24, 27, and 33) were excluded from the malignancy analysis because of the lack of a definitive diagnosis. Of those three patients, one declined further investigation of the lung lesion, one (with advanced colorectal adenocarcinoma) was lost to follow-up, and the other (with prostate adenocarcinoma and esophagogastric transition adenocarcinoma) is still undergoing follow-up of the lesion after a multidisciplinary decision. Therefore, a total of 36 lesions were analyzed: 19 (52.8%) in the malignant group and 17 (47.2%) in the benign group. Of the 19 malignant lesions, five (26.3%) were carcinoid tumors, accounting for 13.9% of the 36 lesions.

Only two of the malignant lesions did not have a histological diagnosis, and both of the affected patients died. The first (case 1) was diagnosed as renal neoplasia and multiple growing pulmonary nodules (interpreted as metastases after multidisciplinary discussion). The other (case 10) had a lesion suspicious for primary pulmonary neoplasia, with mediastinal lymph node enlargement, together with bone, brain, and adrenal lesions suggestive of metastasis. Unfortunately, in the latter case, it was not possible to perform a biopsy because of the clinical status of the patient, who died hours after the procedure was attempted.

**Figure 3:**
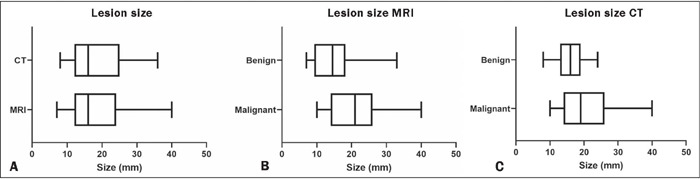
Box plots of lesion sizes showing measurements by CT and MRI **(A),** as well as the differences between the benign and malignant groups, by MRI **(B)** and by CT **(C).**

On HR T2 sequences, no statistically significant difference was found between benign and malignant lesions in terms of the mean signal intensity (*p* = 0.1196), SDs (*p* = 0.7525), or image quality measured by the signal-to-noise ratio (*p* = 0.8056). However, there was a statistically significant difference in the dimensions of the lesions between the groups, the mean lesion size being 21 ± 7.8 mm in the malignant group; and 15 ± 7.1 mm in the benign group (Figure 3).

**Figure 4:**
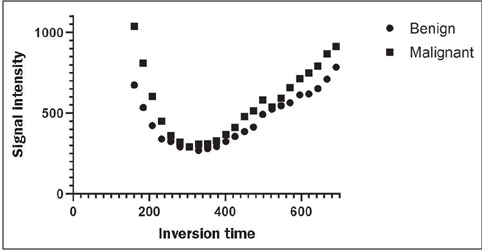
Distribution of the mean signal intensity in the benign and malignant groups at the various inversion times on LL T1 relaxometry sequences.

**Figure 5:**
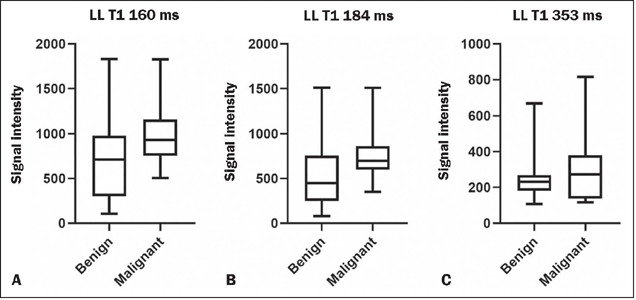
Box plots of the mean signal intensity on LL T1 relaxometry sequences at inversion times of 160 ms **(A),** 184 ms **(B)** and 353 ms **(C),** by group.

**Figure 6:**
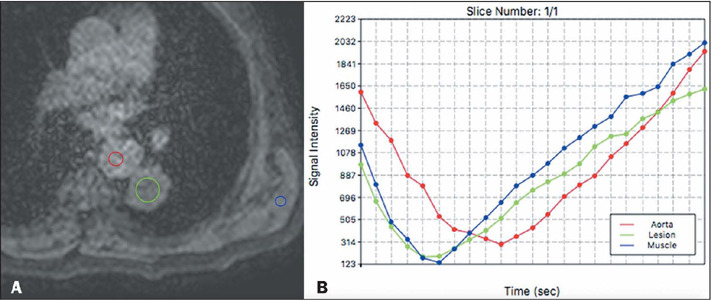
LL T1 relaxometry se-quence at an inversion time of 160 ms **(A)** and a line graph showing the signal intensity for each region of interest at different inversion times **(B).**

The mean T1 relaxometry values did not show a statistical difference between the groups (*p* = 0.3006); however, in the graphical evaluation of the distribution of the mean of the signal intensity values measured at the different inversion times in the LL T1 relaxometry sequence, it was possible to observe a discrete difference between the two groups at the extremities of the inversion times studied (Figure 4). This subanalysis of the individual inversion times showed a statistically significant difference at the shortest inversion time (160 ms), as illustrated in Figures 5 and 6. At the other inversion times, there were no statistical differences between the groups, with *p*-values ranging from 0.0715 (for 184 ms) to 0.9671 (for 498 ms).

**Figure 7:**
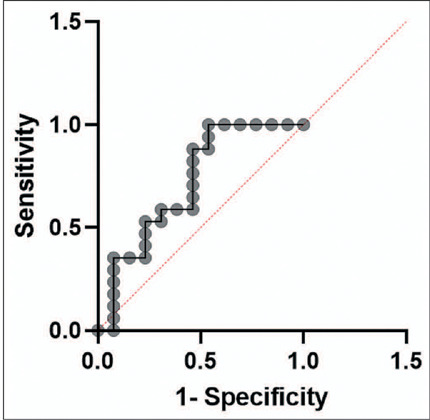
Receiver operating characteristic curve of the mean signal intensity of an FLL measured at an inversion time of 160 ms in an LL T1 relaxometry sequence.

In a receiver operating characteristic curve analysis for the LL T1 relaxometry sequence at the inversion time of 160 ms (Figure 7), we found an area under the curve of 0.71 (95% confidence interval [CI]: 0.52–0.91). That translates to a signal intensity above 1,102 having a specificity for malignancy of approximately 92% (95% CI: 0.67–0.99). Therefore, signal intensity measured at the inversion time of 160 ms helped to distinguish between malignant and benign lesions in the LL T1 relaxometry sequence (*p* < 0.05).

In the analysis of the complete MRI examination, including all sequences, none of the malignant lesions were mischaracterized as benign. The specificity of that analysis was 68.7%, with a positive predictive value of 79.2% and an accuracy of 85.7%. Interobserver agreement in the MRI assessment was fair (kappa = 0.351), with a SD of 0.154 (95% CI: 0.050-0.652).

The CT categorization of lesions as benign or malignant had a sensitivity of 94.7% and a specificity of 50.0% (with a positive predictive value of 69.2%, negative predictive value of 69.2%, and accuracy of 74.3%). The in-terobserver agreement on CT was substantial (kappa = 0.612), with a SD of 0.157 (95% CI: 0.305–0.919).

There was no statistically significant difference between CT and MRI in terms of the measurements of the lesion dimensions (Figure 3). Morphologically, all of the lesions were classified as either nodules or masses in both analyses. The sample included only one partially solid lesion, one with a pseudocavity, and two with air bronchograms, all identified by both imaging modalities (Table 3). In the characterization of pleural tags, CT proved superior, identifying a total of 13 cases, whereas only seven were identified on MRI. No statistically significant differences were observed between the two modalities in the characterization of the nature, type, or contours of the lesions (Table 4).

**Table 4:** Location and morphological features of FLLs identified with both imaging modalities

Feature	CT	MRI
Lung segment (right/left), n/n		
I/I + II	2/3	3/5
II	4/-	4/-
III	3/2	2/2
IV	0/1	0/1
V	2/1	3/0
VI	4/3	2/1
VII/VII+VIII	0/1	0/2
VIII	2/-	2/-
IX	0/2	0/1
X	3/1	3/3
Central or hilar	0/1	1/0
Nature, n		
Solid	34	34
Ground-glass	0	0
Partially solid	1	1
Type, n		
Nodule	32	32
Mass	3	3
Consolidation	0	0
Other	0	0
Contours, n		
Regular	24	24
Lobulated	5	4
Spiculated	5	3
Irregular	1	4

## DISCUSSION

There is a high prevalence of FLLs on chest imaging. Despite the chance that an FLL will be malignant, it represents a benign finding in most cases. The benefit of diagnosing lung cancer at the initial stages is that it provides a significant improvement in the prognosis (five-year disease-free survival and overall mortality). In clinical practice, it is not common to employ MRI for the characterization of FLLs. However, there is still a great potential for developing new techniques that could add value to its role; sequences such as HR T2 and LL T1 relaxometry have the potential to do so. In this prospective, original study, we have shown that it is possible to evaluate FLLs with those sequences, the HR T2 sequence aiding in the morphological evaluation and the LL T1 relaxometry sequence aiding in the quantitative evaluation. The main problems in the acquisition of those sequences were related to errors in the localization of the FLLs, showing that it may be necessary to have a physician in the examination room during the programming of such sequences. It is noteworthy that this additional characterization of lung lesions by these sequences does not require the use of sophisticated equipment or drugs, which may be unavailable in suburban and rural areas.

There is currently concern about the permanent deposition of gadolinium—the basis of the main MRI contrast media—in tissues, even in patients who do not have nephropathy. Although no diseases have been linked to such deposition, the hypothetical harm, the discomfort caused by the venipuncture, and the potential for puncture complications have prompted initiatives to develop new, contrast-free technologies^(27,28,29)^. That justifies the search for alternatives with the potential to add information and diagnostic confidence in situations in which the injection of contrast medium is contraindicated. Depending on how this evolution occurs, we may see a day when contrast injection is unnecessary, which would reduce the time to prepare patients, as well as reducing costs, including those related to renal function testes and to the contrast medium itself. The HR T2 and LL T1 relaxometry sequences studied here do not require the use of contrast media.

In our study sample, the LL T1 relaxometry and HR T2 sequences did not add accuracy for distinguishing between benign and malignant lesions. However, that was a secondary, limited analysis in our study, given the small number of lesions evaluated, which could be refined in larger, multicenter studies. Nevertheless, signal intensity measured at the first inversion time of the LL T1 relaxometry sequence (160 ms) helped to distinguish between malignant and benign lesions.

Qualitative evaluation of the signal on MRI has good diagnostic capability, which can be refined by quantitative analysis. However, unlike CT images^(30)^, at the output of the image reconstruction algorithm in MRI, an absolute measure of the signal of an organ has no direct correlation with its constitution or physical properties, because each image has its signal intensity scale suited to the anatomical structures considered. Therefore, signal intensity is more difficult to use as a quantitative parameter when analyzing MRI scans. When characterizing the signal of an FLL, the intensity measured in the images before intravenous contrast medium injection may be greater than the intensity measured from the contrast-enhanced images, even if it is not qualitatively apparent. When analyzing MRI scans, it is always necessary to keep in mind the fact that the signal is a relative quantity with its matrix scaled within a limited gray scale. Therefore, for quantitative MRI evaluation, advanced techniques such as LL T1 relaxometry are required. However, with the popularization of cardiac MRI examinations in recent years, LL T1 relaxometry has become reasonably available, facilitating its use in the study of FLLs without a relevant increase in costs, because it requires no equipment or software upgrades. As a counterpoint, the sequence has limitations in the setting of high heart rates, the motion of the lesions, and the potential for unsatisfactory mathematical fitting at processing. It is also important to remember that T1 relaxometry performed by other techniques can provide different results.

Studies of T1 relaxometry in the lung are scarce, with few showing its applicability in parenchymal disease^(31,32)^. To our knowledge, there have been no studies assessing the ability of T1 relaxometry to characterize FLLs. The subanalysis of the different inversion times in the LL T1 relaxometry sequence showed the potential to add accuracy in distinguishing benign and malignant lesions.

One of the strengths of MRI lesion assessment is the ability to assess “texture”, due to the high tissue contrast of the method. This allows the data in the images to be translated into subjective characteristics (in the radiological visual assessment in clinical practice) or even into infinite mathematical parameters, allowing for correlation with clinical and even genomic data in the current concept of radiomics. In this field of study, in which a massive amount of data is extracted from the different gray tones of the image pixels, it is possible to increase the accuracy of the methods by improving the quality of the images.

Conventional MRI has greater specificity for the detection of regional invasion than does CT^(33)^. With the HR T2 images, there is potential to improve that specificity. By having a lower signal-to-noise ratio than conventional T2 images, HR T2 images may be of lower quality. However, MRI quality assessment is complex, and other parameters must be considered in this assessment. For example, the HR T2 sequence was considered the most accurate to characterize relevant findings in an FLL, such as pseudocavities and pleural tags, which are morphologic features that are known to favor malignancy. The prevalence of lesions with these secondary characteristics was low in the sample, and CT proved superior to MRI for the characterization of pleural tags. One possible explanation for this might be the greater slice thickness used in the MRI examinations, leading to partial volume effects that hindered their characterization.

Despite the recognized role of HR T2 sequences in evaluating pathologies of the central nervous system^(34)^ and pelvis^(19,20,35,36)^, few studies have evaluated their use in the thorax, often being limited to works with animal cadavers^(37)^ because of the potential artifacts generated by respiratory movements. There are now MRI techniques that we can use to reduce such image quality detractors, allowing a precise characterization of FLLs.

In line with the Fleischner Society statement regarding MRI use, our study demonstrated that MRI evaluation had better accuracy than did CT (86% vs. 74%). In addition, all of the lesions categorized as malignant by MRI were subsequently also categorized as malignant by pathology (i.e., MRI did not mischaracterize any malignant lesions as benign).

By using techniques widely available at most centers, integrating the HR T2 and LL T1 relaxometry sequences into routine clinical practice could improve the diagnostic performance of chest MRI at a low cost, although it requires careful planning and consideration. Initially, the focus should be on training radiologists and MRI technologists, to ensure that they are proficient in acquiring and interpreting these specific sequences. In addition, developing standardized protocols for the use of these sequences in evaluating FLLs will be essential. Collaboration with multidisciplinary teams, including oncologists, thoracic surgeons, and radiologists, will facilitate seamless integration and optimize case management. This could lead to a reduction in the number of unnecessary procedures, such as biopsies and even some surgeries, thus lowering overall health care expenditures. Furthermore, if these techniques can provide detailed imaging without the use of contrast agents, there could be additional cost savings and a reduction in the risks associated with the use of contrast media, particularly in patients with preexisting renal conditions. However, a thorough cost-benefit analysis will be essential for health care institutions considering the adoption of these advanced MRI techniques, contingent upon further validation.

### Study limitation

Our study has some limitations. First, the relatively small sample size limits the generalizability of our findings and may not capture the full variability of FLL characteristics. In addition, the potential selection bias, particularly due to the inclusion of patients with atypical imaging findings, might have been responsible for the high proportion of benign and carcinoid lesions observed. Furthermore, the complexity of the MRI acquisition protocols and the need for precise lesion localization introduced technical challenges, which resulted in some data being excluded from the analysis. The number of lesions lost through poor localization shows that the acquisition planning of the sequence can be complex for technicians, analogous to that of spectroscopy.

Although T1 relaxometry can effectively be performed with other MRI sequences, there have been no studies dedicated to its standardization and to evaluating its role in the characterization of FLLs. Moreover, the values of native T1 vary between different scanners; therefore, multicenter studies are needed in order to refine our findings. To mitigate these limitations in future studies, we recommend conducting larger-scale investigations with more diverse patient populations. Multicenter trials would be particularly valuable in validating our findings and ensuring the standardization of MRI protocols across different institutions. Advances in MRI technology, such as thinner slices and better synchronization techniques, should be explored to reduce technical errors and enhance image quality. By addressing these limitations, future research could build on our findings and further establish the utility of HR T2 and LL T1 relaxometry sequences in the evaluation of FLLs.

## CONCLUSION

In conclusion, both of the MRI sequences proposed were feasible in the evaluation of FLLs. A specific analysis of the LL T1 relaxometry sequence may help distinguish between benign and malignant lesions, and the HR T2 sequence seems to be the best sequence for evaluating certain morphological characteristics.
